# Phylogeny of Maleae (Rosaceae) Based on Complete Chloroplast Genomes Supports the Distinction of *Aria*, *Chamaemespilus* and *Torminalis* as Separate Genera, Different from *Sorbus* sp.

**DOI:** 10.3390/plants10112534

**Published:** 2021-11-21

**Authors:** Bartosz Ulaszewski, Sandra Jankowska-Wróblewska, Katarzyna Świło, Jarosław Burczyk

**Affiliations:** Department of Genetics, Faculty of Biological Sciences, Kazimierz Wielki University, Chodkiewicza 30, 85-064 Bydgoszcz, Poland; ulaszewski@ukw.edu.pl (B.U.); Jankowska-wroblewska@ukw.edu.pl (S.J.-W.);

**Keywords:** phylogenomics, complete chloroplast genome, wild service tree

## Abstract

Several genera formerly contained within the genus *Sorbus* L. *sensu lato* have been proposed as separate taxa, including *Aria*, *Chamaemespilus* and *Torminalis*. However, molecular evidence for such distinctions are rather scarce. We assembled the complete chloroplast genome of *Sorbus aucuparia*, another representative of *Sorbus* s.s., and performed detailed comparisons with the available genomes of *Aria edulis*, *Chamaemespilus alpina* and *Torminalis glaberrima*. Additionally, using 110 complete chloroplast genomes of the Maleae representatives, we constructed the phylogenetic tree of the tribe using Maximum Likelihood methods. The chloroplast genome of *S. aucuparia* was found to be similar to other species within Maleae. The phylogenetic tree of the Maleae tribe indicated that *A. edulis*, *C. alpina* and *T. glaberrima* formed a concise group belonging to a different clade (related to *Malus*) than the one including *Sorbus* s.s. (related to *Pyrus*). However, *Aria* and *Chamaemespilus* appeared to be more closely related to each other than to *Torminalis*. Our results provide additional support for considering *Aria*, *Chamaemespilus* and *Torminalis* as separate genera different from *Sorbus* s.s.

## 1. Introduction

The family Rosaceae Juss. consists of about 6000 species classified in 120 genera (Catalogue of Life; https://www.catalogueoflife.org/ [1], accessed on 17 May 2021). However, phylogenetic relationships within Rosaceae are problematic due to considerable diversity in morphology and the possibility for hybridization and apomixes among the species [2,3]. There have been numerous studies examining relationships in the Rosaceae family [3]; however, it is important to resolve the phylogeny at lower taxonomic levels at a finer scale [4].

The tribe Maleae consists of shrubs and small trees and is of great economic and ecological importance [4]. It consists of about 1200 species in nearly 40 genera (depending on classification); however, 21 genera are represented by fewer than 10 species each, including 11 monospecific genera. *Sorbus* L. is among the genera incorporating a considerable number of species. Formerly, *Sorbus sensu lato* included several species currently distinguished as separate genera (e.g., *Aria* (Pers.) Host, *Chamaemespilus* Medik., *Cormus* Spach, *Torminalis* Medik.) [5,6,7]. However, in many research papers, dated taxonomical names are commonly used [8], which confounds the Maleae phylogeny. Therefore, the goal of this paper was to resolve the phylogenetic structure of the Maleae tribe by using new molecular evidence.

Since 2007, DNA sequencing has been used to guide the phylogenetic reconstruction in an attempt to clarify the status of the Malae group. [2,5,6,7,9]. In particular, chloroplast genome sequences are useful in generating molecular phylogenetic relationships as they are generally free from paralogues, have a moderate size and relatively low nucleotide substitution rates. However, the possibility of chloroplast capture, i.e., the sharing of chloroplast genomes among hybridized species [10,11], may complicate the inference of phylogeny.

The three genera distinguished from *Sorbus* s.l., namely *Aria*, *Chamaemespilus* and *Torminalis*, usually form a clade separate from *Sorbus sensu stricto* [2,4]. However, their phylogenetic position relative to other representatives of Maleae has rarely been investigated in detail [3,7,12]. The existing studies have either used partial cpDNA regions [4,9], or the analyses were confounded by use of dated taxonomic nomenclature [8].

In this study, we present a new assembly of the complete chloroplast genome of *Sorbus aucuparia* L., another representative of *Sorbus* s.s. Given the availability of complete chloroplast genomes of several Maleae species assembled recently by various research groups [8,13,14,15,16,17,18], we attempted to reconstruct the phylogeny of the Maleae tribe based on complete chloroplast genomes, with a special focus on the relationships among the genera formerly assigned to *Sorbus* s.l.

## 2. Results

### 2.1. Chloroplast Genome of Sorbus aucuparia

**Figure 1:**
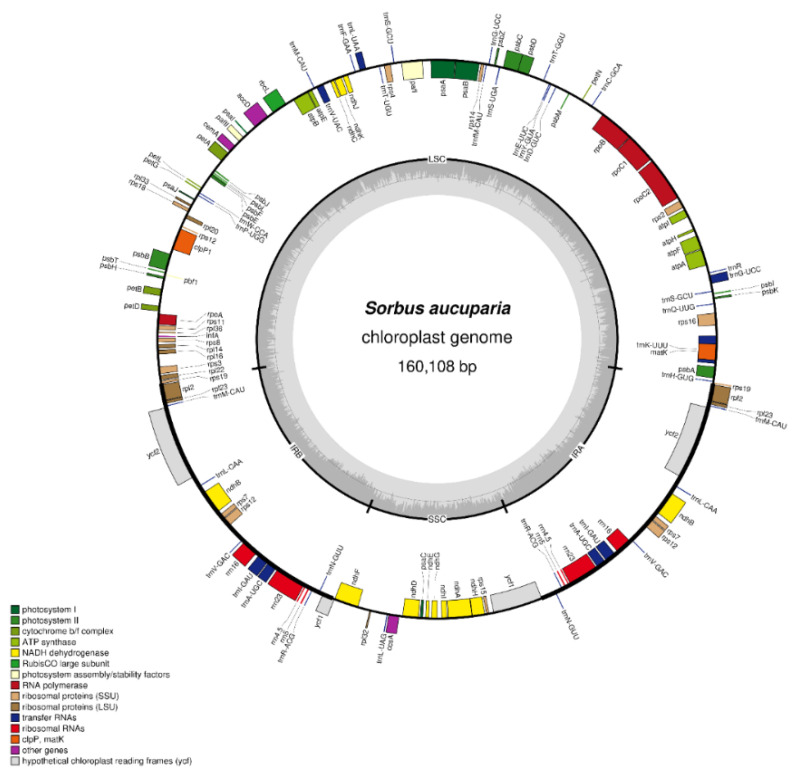
Chloroplast gene map of *Sorbus aucuparia*. Genes are grouped in categories highlighted in various colors. If a gene is transcribed clockwise it is shown on the inside of outer circle; if transcribed counter-clockwise it is shown on the outside of outer circle. The inner circle shows genome regions: LSC—large single-copy; SSC—small single-copy; IR(A/B)—inverted repeat.

The de novo assembly of *Sorbus aucuparia* chloroplast genome is a typical 160,108 bp long quadripartite structure consisting of an 88,214 bp large single copy (LSC) region and a 19,506 bp small single copy (SSC) region, and two 26,194 bp inverted repeat regions (Figure 1). The genome consists of 130 genes in total, out of which we identified 85 protein-coding genes, 37 tRNA genes and 8 rRNA genes (Table S1).

### 2.2. Phylogeny of Maleae Tribe Based on Complete Chloroplast Genomes

**Figure 2:**
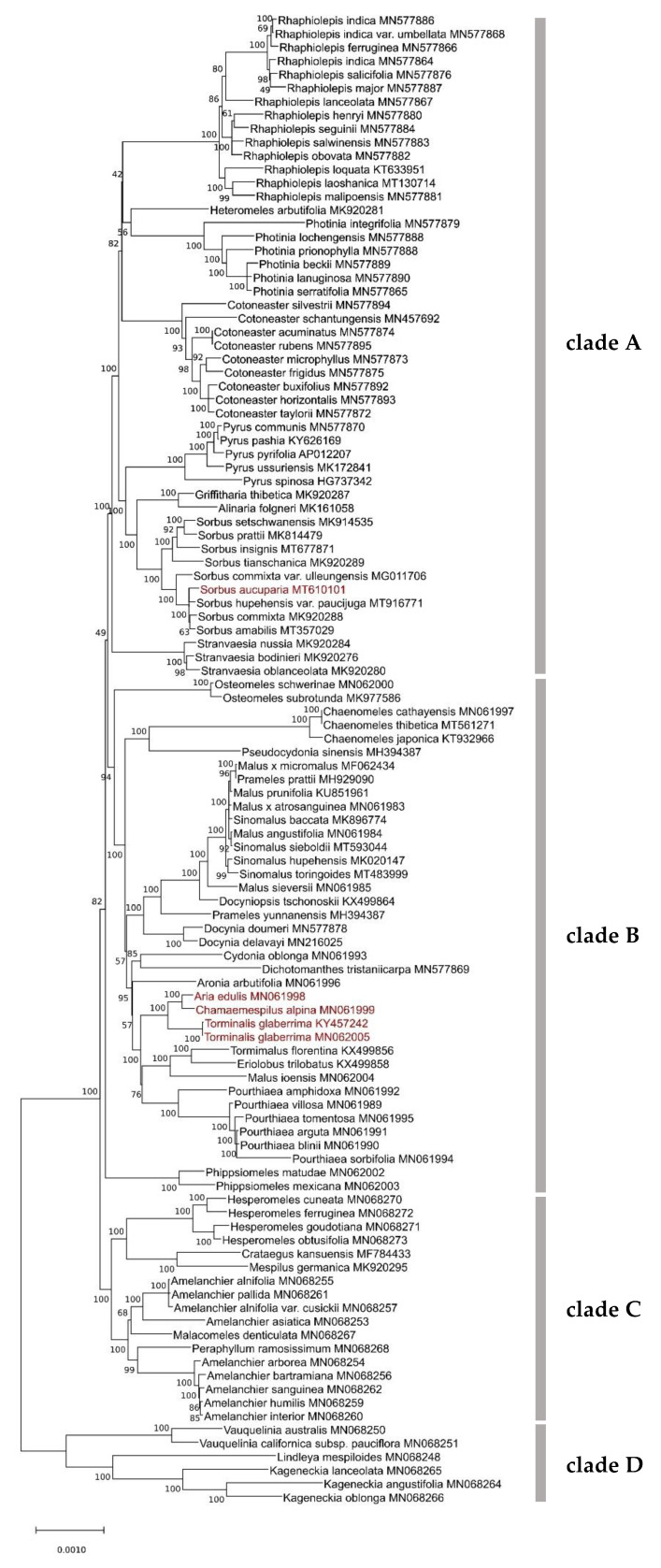
Phylogenetic tree of Maleae tribe reconstructed based on complete chloroplast genome of 110 taxa representing 36 genera inferred based on the Maximum Likelihood method. Numbers indicate the percentage of bootstraps supporting the clade.

The phylogenetic tree with the highest log likelihood (−288,538.88) estimated based on the Maximum Likelihood method for 110 complete chloroplast genomes representing 36 genera is shown in Figure 2. The percentage of trees in which the associated taxa clustered together is shown next to the branches. There were a total of 145,424 nucleotide positions in the final dataset; however, about 47.46% of sites appeared to be evolutionarily invariable in the investigated dataset. All positions containing gaps and missing data were eliminated (complete deletion option).

Four well-supported clades (A–D) could be resolved in the phylogenetic tree of the Maleae tribe (Figure 2). Clade A included *Rhaphiolepis*, *Heteromeles*, *Photinia*, *Cotoneaster*, *Pyrus*, *Griffitharia*, *Alinaria*, *Sorbus* and *Stranvaesia*. The most diverse clade B included *Osteomeles*, *Pourthiaea*, *Malus*, *Tormimalus*, *Eriolobus*, *Aria*, *Chamaemespilus*, *Torminalis*, *Aronia*, *Cydonia*, *Dichothomanthes*, *Chaenomeles*, *Pseudocydonia*, *Docynia*, *Prameles*, *Sinomalus* and *Phippsiomeles*. Clade C involved *Hesperomeles*, *Crataegus*, *Mespilus*, *Amelanchier*, *Malacomeles* and *Peraphyllum*. Finally, the most divergent clade D included *Vaquelinia*, *Lindleya* and *Kageneckia* (Figure 2).

Several genera represented by more than one species, given the list of species used in our analyses, appear to form monophyletic groups. This includes *Rhaphiolepis*, *Photinia*, *Cotoneaster*, *Pyrus*, *Sorbus*, *Stranvaesia*, *Osteomeles*, *Pourthiaea*, *Chaenomeles*, *Docynia*, *Phippsiomeles*, *Hesperomeles*, *Vauquelinia* and *Kageneckia* (listed from top to bottom of the diagram presented in Figure 2). Notably, *Sorbus* species form a well-supported clade related to *Pyrus*. On the other hand, the three species that are of special interest in this study: *A. edulis*, *C. alpina* and *T. glaberrima* form a distinct clade sister to *Pourthiaea* clade and the multispecific clade composed of *Malus ioensis*, *Tormimalus florentina* and *Eriolobus trilobatus*. It is worth noting that the chloroplast genomes of two individuals of *T. glaberrima*, assembled by different research teams, clustered together on the phylogenetic tree.

Besides the three species of special interest in this study, there were several other genera represented by single species. *Heteromeles arbutifolia* was found to be related to *Photinia*. *Griffitharia thibetica* and *Alinaria folgneri* clustered together among the *Pyrus* and *Sorbus* clades. *Crataegus kansuensis* and *Mespilus germanica* formed a clade sister to *Hesperomeles*. *Malacomeles denticulata* and *Peraphyllum ramosissimum* appeared to be located within the clade of *Amelanchier*.

Intriguingly, clade B was the most diverse with 19 genera. Some members of *Malus* and *Prameles* were separated between different sub-clades and some members of *Malus* and *Sinomalus* formed the same clade.

### 2.3. Detailed Comparative Analysis of Four Chloroplast Genomes

Detailed comparisons between the chloroplast genomes of four species, *Sorbus aucuparia* (MT610101), *Torminalis glaberrima* (KY457242)*, Aria edulis* (MN061998) and *Chamaemespilus alpina* (MN061999), revealed variations across the whole genome and in all basic assembly elements (LSC, SSC and IR), although the composition and order of genes and other annotated elements remained the same (Table S2; Figure S1).

The cpDNA of newly assembled *S. aucuparia* was found to be the smallest (160,108 bp) among the four species; however, the different sections of the chloroplast genome appeared to have minor differences. The four investigated species have the same numbers of protein-coding, tRNA and rRNA genes, and pseudogenes (Table S2).

A more detailed investigation of alignments between the four genomes shows that the majority of differences are located in non-coding regions. Between these four species, six possible pairwise alignment were generated (Table 1). The total number of non-matching nucleotides ranged from 560 for the *A. edulis* and *C. alpina* pair, to 3004 for the *S. aucuparia* and *T. glaberrima* pair. Indels were the dominant source of pairwise differences (80.4 –88.7%). Interestingly, the proportion of nucleotide substitutions was larger when pairwise comparisons involved *S. aucuparia*. Only 11 mismatches were found in coding regions of *A. edulis* and *C. alpina*, about 60 when *T. glaberrima* was compared to *A. edulis* or *C. alpina*, but the largest number of mismatches in coding regions (107–124) were noticed when pairwise comparisons involved *S. aucuparia* (Table 1).

## 3. Discussion

Phylogenomics based on complete chloroplast genomes is increasingly becoming the most attractive means of obtaining initial insight into the phylogeny of several plant taxa [19,20,21]. However, for this purpose complete and well-assembled chloroplast genomes of the species of interest are needed. Fortunately, due to advances in next-generation sequencing, and bioinformatic assembly approaches, a large number of newly developed chloroplast genomes have become available. The new assembly of the chloroplast genome of *S. aucuparia* obtained in this study extends the list of the available cpDNA genomes of the species belonging to the Maleae tribe, enabling a closer look into its phylogeny. The chloroplast genome of *S. aucuparia* assembled in this study is similar to other chloroplast genomes of the representatives of the Maleae tribe in terms of genome size, quadripartite structure and gene content [8,13,15,17].

Chloroplast phylogenomic analyses within the Maleae tribe members are difficult because this tribe seems to be less diverse compared to other taxonomic groups within Rosaceae [3]. In general, our cpDNA-exclusive phylogeny is similar to the Maleae phylogeny presented by Zhang et al. [3]. It is comparable to the phylogeny constructed by using chloroplast and ITS sequences, as well as with phenotypic characteristics presented by Lo and Donoghue [12] (Figure 4 in [12]). The phylogenetic position of several genera investigated in this paper (Figure 2) are also in line with the results of previous studies. For example, *Rhaphiolepis* (following refinement by Liu et al. [22]), *Heteromeles*, *Photinia* (updated by Liu et al. [17] and *Cotoneaster* form the concise group located within clade A, which has been previously demonstrated by other authors [8]. The complex clade C involving *Hesperomeles*, *Crataegus*, *Mespilus*, *Amelanchier*, *Malacomeles* and *Peraphyllum* appeared to be well resolved and positioned as basalmost compared to clades A and B, similarly as in previous studies [8,17]. *Vauquelinia*, *Lindleya* and *Kageneckia* formed a well-supported clade D (Figure 2), quite distant from other Maleae species [8,12].

Finally, clade B appeared to be the most complex and challenging in the context of the genus *Malus*. *Tormimalus florentina* was earlier classified as belonging to *Malus* s.l. (*Malus florentina*); however, its taxonomical status has been clarified by Holub [23]. *Eriolobus trilobata* and *Docyniopsis tschosnoskii* were wrongly described in the NCBI database with their synonym names as *Malus trilobata* and *Malus tschosnoskii*, respectively (Table S3). The location of *Malus ioensis* on the phylogenetic tree close to *Tormimalus* and *Eriolobus,* raises questions about the origin of that taxon. Recent taxonomic nomenclature changes proposed by Rushforth [7] and accepted in CoL [1], mean that several other species belonging previously to the genus *Malus* appear in this study as *Sinomalus* or *Prameles* (Table S3). Without these changes, the apple clade would appear to be nearly monophyletic. The polyphyletic origin of *Malus* has been reported by several authors [8,17,18]; however, the genus deserves closer attention extending beyond the scope of this study.

The complete chloroplast genome of *T. glaberrima* (synonym name: *Sorbus torminalis*) assembled by our research team [14] was one of the earliest among the representatives of the Maleae tribe, besides the *Malus* and *Pyrus* species. It was already documented to be more closely related to *Malus* than to *Pyrus* or even *Sorbus* s.s. [14,15,18]. Our analysis based on chloroplast genomes fully supports the distinction of *Aria*, *Chamaemespilus* and *Torminalis* as separate genera, quite distinct from the genus *Sorbus* s.s. [5,6,7].

Among the three genera of special interest in this study, *Aria* and *Chamaemespilus* seem to be more closely related. This is supported by their close location on the phylogenetic tree (Figure 2) and is also corroborated by the lowest number of pairwise mismatches (560 bp) with only 11 (1.8%) mismatches in coding sequences between their chloroplast genomes (Table 1). The pairwise comparisons of these two species with *T. glaberrima* indicated about 1900 mismatched bp, with about 60 (2.6%) of them located in coding sequences and, thus, corresponds with its basal location to *Aria* and *Chamaemespilus* on the phylogenetic tree (Figure 2). However, the mean genetic distance among the chloroplast genomes of the three species seems to be low (6.72 × 10^−4^), as compared, for example, to the mean genetic distance within the genus *Sorbus* s.s. (7.38 × 10^−4^) (Table S4), thus, somehow questioning the genus status of *Aria*, *Chamaemespilus* and *Torminalis* and pointing to the need for further research. In the end, the clarification of the *Aria*, *Chamaemespilus*, and *Torminalis* phylogenetic position supports the monophyly character of the genus *Sorbus*. Although our investigation involved only a handful of *Sorbus* s.s. species [24], they formed a concise clade as previously seen in other studies [24].

However, some discrepancies between nuclear and chloroplast-based phylogenies are common in plants, including Rosaceae [3,12,25,26]. While not investigated in this study, the phylogenetic position of *Aria*, *Chamaemespilus* and *Torminalis* based on nuclear DNA sequences seems to be not fully resolved. For example, Liu et al. [17], using nrDNA data, located these three species within the same clade but jointly with *Sorbus thibetica*. In another study, the same clade included also *Pyrus communis* [8]. Xiang et al. [25], based on 113 nuclear genes, found *Sorbus aria* (synonym of *Aria edulis*) to be closely related with other *Sorbus* species. Interestingly, Lo and Donoghue [12] suggested that *Micromeles* (synonym of *Alinaria*) resulted from hybridization between *Aria* and *Sorbus* s.s., thus pointing to their close relationship. We believe that a more detailed picture of the phylogenetic relationships between *Sorbus*, *Torminalis* and other *Maleae* genera should soon be available given the upcoming complete nuclear genomes of several taxa assembled, among others, by our research team.

## 4. Materials and Methods

### 4.1. De Novo Assembly of the Chloroplast Genome of Sorbus aucuparia

In this paper, we first assembled the complete chloroplast genome of *Sorbus aucuparia* L., the representative of *Sorbus* s.s. The material used for chloroplast genome assembly of *S. aucuparia* was collected in spring 2019 from a > 50-year-old individual (Figure S2), located in a Tryszczyn Forest Nursery, Poland (53.171761 N, 17.941530 E). The DNA was isolated from leaves using a protocol described in Wang et al. [27]. Genomic library construction (TruSeq DNA PCR-Free, 350-bp insert; Illumina, USA) and sequencing on NovaSeq 6000 device (Illumina, USA) was outsourced to Macrogen Inc. (Republic of Korea). The sequencing generated 646 million paired-end 151-bp reads.

Chloroplast genome was assembled de novo with 10% of randomly selected primary Illumina reads using NOVOPlasty v 4.1. [28] and rbcL sequence (KM360990) of *Sorbus domestica* as seed. This generated a 160,108 bp circular genome with 8688x coverage. Ambiguous nucleotides were manually corrected with the assistance of bwa-mem [29] for Illumina read mapping to the obtained genome and Tablet [30] for results visualization. The annotation was conducted with GeSeq ChloroBox [31] using chloroplast genomes of *Sorbus commixta var. ulleungensis* (MG011706), *Sorbus prattii* (MK814479) and *Sorbus tianschanica* (MK920289) as references. The annotated assembly of the chloroplast genome of *S. aucuparia* was uploaded to GenBank (MT610101).

### 4.2. Phylogeny of the Maleae Tribe Based on Complete Chloroplast Genomes

The available complete chloroplast genomes of Maleae species were obtained from the NCBI website. The complete list of the species used in this study and relevant chloroplast genome accession numbers are enclosed in Table S3. However, because there were some discrepancies in taxon names between NCBI and Catalogue of Life (CoL) [1] databases, we finally used the names of accepted species as they appear in CoL [1], with few exceptions. Recently, some authors have provided the revision of *Photinia* complex based on complete chloroplast genomes and nrDNA sequences, [17] where some species considered previously as *Photinia* were promoted to already existing genus *Stranvaesia Lindl*., but some others were assigned to the newly proposed genus *Phippsiomeles* [17]. The list of species names is included in Table S3. In total, 110 complete chloroplast genomes originating from 36 genera were used for phylogeny reconstruction of the Maleae tribe. This included the chloroplast genome of *Sorbus aucuparia* developed in this study.

Nucleotide sequences of the complete chloroplast genomes were downloaded as FASTA files, which were then assembled into one large multi-FASTA file. All sequences were adjusted to start with the sequence GGGCGAACGACGGGAATT…… (74 bp), which was found identical (monomorphic) among all investigated sequences. This sequence is a part of *trnH-GUG* gene, and is considered highly conservative among plants species [32].

Complete chloroplast genomes were aligned using MAFFT v 7 online server [33] with default settings. The aligned sequences were stored in a FASTA file and then converted to MEGA format using MEGA X [34]. The phylogeny was inferred in MEGA X by using the Maximum Likelihood method, with complete deletion option. General Time Reversible model (GTR+G+I) [35] was selected as the best substitution model based on Akaike’s information criterion (AIC) with MEGA X [34]. The initial tree for the heuristic search was obtained automatically by applying Neighbor-Join and BioNJ algorithm to a matrix of pairwise distances estimated using the Maximum Composite Likelihood (MCL) approach and then selecting the topology with a superior log likelihood value. Confidence of phylogenetic tree was tested based on 100 bootstraps. The tree was drawn to scale, with branch lengths measured in the number of substitutions per site.

### 4.3. Detailed Comparative Analysis of Four Chloroplast Genomes

We re-annotated the assemblies of *S. aucuparia* (MT610101), *T. glaberrima* (KY457242), *A. edulis* (MN061998) and *C. alpina* (MN061999) using GeSeq ChloroBox as mentioned in the previous paragraph, to avoid annotation errors. Synteny comparisons were performed with Mauve software [36]. Detailed lookup of the differences between the assemblies was carried out by aligning them with MAFFT [37], the results of this procedure were obtained with UniPro UGENE [38].

## 5. Conclusions

The newly assembled chloroplast genome of *S. aucuparia* is similar to the chloroplast genomes of other representatives of the Maleae tribe in terms of its size, structure and gene content. The phylogenetic tree based on complete chloroplast genomes of several taxa of Maleae indicated that *Sorbus* s.s. and the three species of interest belong to different clades; however, *Aria*, *Chamaemespilus* and *Torminalis* appeared to be closely related to each other. Detailed comparisons between *S. aucuparia* and the three species *Aria edulis*, *Chamaemespilus alpina* and *Torminalis glaberrima*, formerly considered as subgenera of *Sorbus* s.l., confirmed that they should be regarded as separate taxa.

## Figures and Tables

**Table 1:** General statistics of non-matching nucleotides in the pairwise alignments of chloroplast genome assemblies of *S. aucuparia, T. glaberrima, A. edulis* and *C. alpina*.

		*S. aucuparia* vs.			*T. glaberrima* vs.		*A. edulis* vs.
		*T. glaberrima*	*A. edulis*	*C. alpina*	*A. edulis*	*C. alpina*	*C. alpina*
*Type*	*Total non-matching sites [bp]*	3004	2673	2788	1923	1855	560
	*Indels [bp]*	2434	2128	2242	1706	1636	486
		(81.0%)	(79.6%)	(80.4%)	(88.7%)	(88.2%)	(86.8%)
	*Substitutions [bp]*	570	545	546	217	219	74
		(19.0%)	(20.4%	(19.6%	(11.3%)	(11.8%)	(13.2%)
	*Coding [bp]*	124	107	109	60	62	11
		(4.1%)	(4.0%)	(3.9%)	(2.5%)	(2.8%)	(1.8%)
*Location*	*Non-coding [bp]*	2880	2566	2679	1863	1793	549
		(95.9%)	(96.0%)	(96.1%)	(97.5%)	(97.2%)	(98.2%)

## Data Availability

Newly generated DNA sequences were uploaded to NCBI Gene Bank under accession number MT610101.

## Supplementary Materials

The following are available online at https://www.mdpi.com/article/10.3390/plants10112534/s1, Table S1. Genes annotated in the chloroplast genome of *Sorbus aucuparia*. Table S2. Comparison of chloroplast assemblies of *S. aucuparia, T. glaberrima, A. edulis,* and *C. alpina.* Table S3. List of names of species included in the phylogenetic analyses. Table S4. Genetic distances among 110 taxa inferred based on complete chloroplast genomes. Figure S1. Synteny comparisons of chloroplast genomes: *Sorbus aucuparia* (1), *Torminalis glaberrima* (2), *Aria edulis* (3) and *Chamaemespilus alpina* (4). The chloroplast genome of *Sorbus aucuparia* was used as the reference sequence. Within each of the alignments, local collinear blocks were marked by the same color and connected by lines. Figure S2. About 50-year-old individual of *Sorbus aucuparia* L. sampled in this study, located in Tryszczyn Forest Nursery, Poland (53.171761 N, 17.941530 E).
