# Supplementary material for: Phylogeny of Maleae (Rosaceae) Based on Complete Chloroplast Genomes Supports the Distinction of Aria, Chamaemespilus and Torminalis as Separate Genera, Different from Sorbus sp
Source: Plants (Basel). 2021 Nov 21;10(11):2534. doi: 10.3390/plants10112534 (PMC8618278; doi:10.3390/plants10112534)
Supplement: Supplementary file 1 [file plants-10-02534-s001.zip › Supplementary Tables S1-S2 and Figure S1.pdf]

Table S1. Genes annotated in the chloroplast genome of *Sorbus aucuparia*.

Gene category	Gene name
Photosystem I	*psaA, psaB, psaC, psaI, psaJ*
Photosystem II	*psbA, psbB, psbC, psbD, psbF, psbH, psbI, psbJ, psbK, psbL, psbM, psbN, psbT, psbZ*
Cytochrome b/f complex	*petA, petB, petD, petG, petL, petN*
ATP synthase	*atpB, atpA, atpE, atpF, atpI, atpH*
Photosystem assembly/stability factors	*pafI*
NADH-dehydrogenase	*ndhA, ndhB, ndhC, ndhD, ndhE, ndhF, ndhG, ndhH, ndhI, ndhJ, ndhK*
RubisCO large subunit	*rbcL*
RNA polymerase	*rpoA, rpoB, rpoC1, rpoC2*
Small subunit of ribosome	*rps2, rps3, rps4, rps7*†*, rps8, rps11, rps12*†*, rps14, rps15, rps16, rps18, rps19*
Large subunit of ribosome	*rpl2*†*, rpl14, rpl16, rpl20, rpl22, rpl23*†*, rpl32, rpl33, rpl36*
Transfer RNAs	*trnA-UGC, trnC-GCA, trnD-GUC, trnE-UUC, trnF-GAA, trnfM-CAU, trnG-UCC, trnH-GUG, trnI-GAU, trnK-UUU, trnL-CAA, trnL-UAA, trnL-UAG, trnM-CAU, trnN-GUU, trnP-UGG, trnQ-UUG, trnR-ACG, trnR-UCU, trnS-GCU, trnS-UGA, trnT-GGU, trnT-UGU, trnV-GAC, trnV-UAC, trnW-CCA, trnY-GUA*
Ribosomal RNAs	*rrn4.5*†*, rrn5*†*, rrn16*†*, rrn23*†
Maturase	*matK*
Protease	*clpP*
Other genes	*cemA, ccsA, infA, accD*
Hypothetical chloroplast reading frames	*ycf1, ycf2*†*, ycf4*

† genes with double copies

Table S2. Comparison of chloroplast assemblies of *S. aucuparia, T. glaberrima, A. edulis,* and *C. alpina.*

	S. aucuparia	***T. glaberrima***	***A. edulis***	***C. alpina***
Length	160,108	160,379	160,222	160,326
GC %	36.54 %	36.53 %	36.55 %	36.48 %
LSC	88,214	88,029	88,145	88,316
SSC	19,506	19,547	19,291	19,224
IR	26,194	26,407	26,393	26,393
Protein-coding genes	85	85	85	85
tRNAs	37	37	37	37
rRNAs	8	8	8	8
Pseudogenes	2	2	2	2

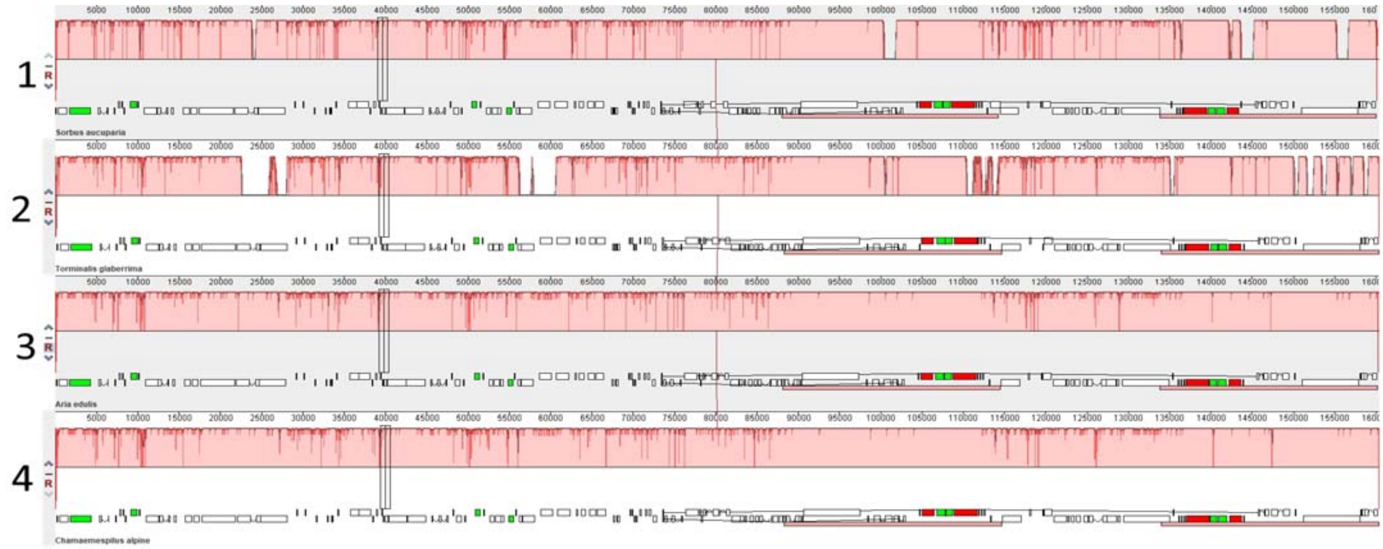

Figure S1. Synteny comparisons of chloroplast genomes: *Sorbus aucuparia* (**1**), *Torminalis glaberrima* (**2**), *Aria edulis* (**3**) and *Chamaemespilus alpina* (**4**). The chloroplast genome of *Sorbus aucuparia* was used as the reference sequence. Within each of the alignments, local collinear blocks were marked by the same color and connected by lines.
